# Anti-Typhoid Inoculation

**Published:** 1902-09-13

**Authors:** 


### ANTI-TYPHOID INOCULATION.

As a general summing up of the present state of knowledge in regard to the anti-typhoid inoculation Professor Wright,[1] of Netley, gives a long series of statistics, the general drift of which is to show that among the inoculated there was a considerable reduction in the incidence of the disease. It would, he says, be illegitimate to state the results in the form of an arithmetical expression representing, as the case might be, the average diminution of the incidence and the average diminution of the death-rate from typhoid fever in the inoculated, for the degree of exposure to infection and the proportion of inoculated to uninoculated are in each case different. But a study of the statistical material does show that in every case except that of the City Imperial Volunteers, and in the case of the staffs of the Portland Hospital and Imperial Yeomanry Branch Hospital at Pretoria, there was at least a twofold reduction in the incidence of the disease among the inoculated. In certain cases, moreover, a far greater reduction in the incidence was achieved—one varying from a sixfold to a twenty-eightfold reduction. Superadded again to the diminished incidence of the disease a striking diminution of case mortality was manifested. "It may be taken that, in the aggregate, the proportion of deaths to cases among the inoculated is approximately half that among the uninoculated." Thus, by combining these observations, it is seen that the united effect of the diminished incidence and the diminished case-mortality manifests itself in a diminished death-rate from typhoid fever among the inoculated. The minimum reduction of death-rate chronicled in the table given is the twofold reduction in the case of the City Imperial Volunteers; but the diminution of death-rate in the case of the inoculated, as shown in the tables, has often exceeded, and has seldom fallen below, a fourfold reduction. On the other hand it is to be noted, as Professor Wright has pointed out on other occasions, that there is a certain risk to be considered in all protective inoculations, the risk to which he particularly refers in this case being that, under certain conditions, inoculation may, for a time at least, leave the system more open to infection at a period when it specially stands in need of protection, this occurring (*a*) in the case where the patient's resistance is naturally low, or has been reduced, as is often the case, by a previous attack of typhoid fever; (*b*) in the case where the patient is inoculated with a full dose of vaccine in actually infected surroundings; and (*c*) in the case where the patient is inoculated with an excessive dose or is re-inoculated too soon.

[1] Lancet, September 6.

